# Exploring the stress sensitization theory with temperamentally inhibited children: a population-based study

**DOI:** 10.1186/s12887-020-02159-w

**Published:** 2020-05-29

**Authors:** Amy Brown, Joanna Bennet, Ronald M. Rapee, Dina R. Hirshfeld-Becker, Jordana K. Bayer

**Affiliations:** 1 School of Psychology and Public Health, La Trobe University, Melbourne, Australia; 2 Centre for Emotional Health, Macquarie University, Sydney, Australia; 3 Child Cognitive Behavioral Therapy Program, Massachusetts General Hospital and Harvard Medical School, Boston, USA; 4 Murdoch Children’s Research Institute, Melbourne, Australia; 5 Department of Paediatrics, The University of Melbourne, Melbourne, Australia

**Keywords:** Child, Internalizing problems, Anxiety, Depression, Life-stressors

## Abstract

**Background:**

This study explored whether temperamentally inhibited children who experience early trauma are vulnerable to developing internalizing problems in the face of later life-stressors.

**Methods:**

A validated screen for temperamental inhibition was distributed to parents of young children attending preschools in six government regions of Melbourne, Australia. Screening identified 11% of children as inhibited (703 of 6347 screened) and eligible for a prevention study. Participants were 545 parents of inhibited preschoolers (78% uptake), of whom 84% were followed into mid childhood (age 7–10 years: wave 1, *n* = 446; wave 2, *n* = 427; wave 3, *n* = 426). Parents and children then completed questionnaires for child internalizing (anxious and depressive) symptoms, and parents received a diagnostic interview for child anxiety disorder. In mid-childhood parents also completed questionnaires annually to describe recent life-stressors experienced by their child, and any potentially traumatic events in the first four years of life.

**Results:**

Only one in 14 temperamentally inhibited children had experienced a potentially traumatic event in early childhood. In mid childhood 56% experienced recent life-stressors. Inhibited children who had early life trauma experienced slightly more anxiety disorder and symptoms in mid childhood. Those children with more recent life-stressors in mid childhood also had slightly more symptoms of anxiety and depression. In contrast to stress sensitization, inhibited children with early trauma plus recent stressors did not show especially high mid-childhood internalizing difficulties.

**Conclusions:**

Early life trauma and recent life-stressors each convey a small risk for children with an inhibited temperament to develop internalizing problems. Nevertheless, early life stress may not always result in negative sensitization for children in the general population.

## Background

Internalizing problems encompass the inner emotional distress of anxiety and depression, which are frequently comorbid [1, 2] and often begin in childhood [3]. To illustrate, Australia’s national Child and Adolescent Survey of Mental Health and Wellbeing found 10% of youth had internalizing problems [4]. Anxiety disorder impacted 7% and major depressive disorder impacted 3% (over half with depression had comorbid anxiety). When internalizing problems develop in childhood they are associated with academic, social, and family dysfunction [5]. Moreover, they can persist into adolescence and adulthood to become long term disorders, with substantial negative life impacts in social, educational, employment and health domains [3, 6, 7]. In pediatric healthcare, understanding the development of internalizing problems in children is important, to inform discussions with families and facilitate early intervention.

Conceptual models include various risk factors and vulnerabilities that can influence the development of internalizing problems in children and youth [8–10]. The premise of these models is that different factors may influence risk at different developmental time points, resulting in the manifestation of internalizing problems from childhood to adulthood. Rapee, Schneiring and Hudson [10] summarised knowledge on key etiological factors for youth anxiety disorders as including genetics, temperament, parenting and individual experiences including adversity. They highlighted limited knowledge about child temperament in relation to mechanisms of environmental factors that requires population-based longitudinal studies. Hankin and colleagues’ [8] review of vulnerabilities and risk mechanisms for youth depression and anxiety similarly highlighted the core roles of child affectivity, information processing biases, and stressors in different domains. They noted that conceptual and empirical work to understand the mechanisms that contribute to youth anxiety/depression are relatively novel in the field with considerable research required in this domain. Extensive childhood research has focused on family factors including parent mental health, parenting (overprotective and/or harsh approaches) [11, 12] as well as genetic inheritance [13, 14]. In sum, research has pointed to the key role of both genetic and environmental factors in the development of anxiety and depression.

Environmental factors such as early childhood traumatic events have been posed in literature as a risk for internalizing problems in youth [15–17]. Trauma is defined as invoking actual or perceived significant harm to an individual or their loved ones [18]. Some examples include vehicular accidents, physical injury, sudden death of a loved one, abuse, and natural disasters. The rate of exposure to such ‘high magnitude events’ for youth has been reported as 25–31% of the population (encompassing childhood and adolescence) [19, 20]. For very young children in highly disadvantaged areas, rates can also be this high with 26% exposed to traumatic events that increase their risk for anxiety [21]. Experiencing traumatic or adverse events in childhood is linked to the development of adolescent and adult psychopathology, including internalizing disorders [22–25]. The neurodevelopmental mechanisms linking adversity to psychopathology include heightened emotional reactivity, threat-related information processing and disruptions in reward processing [26, 27].

In another body of literature broader life-stressors place youth at risk for internalizing problems; events with negative consequences that outweigh a child’s ability to cope but are not considered traumatic [15, 28]. Illustrative examples include parental separation, moving house, or death of a pet. In Costello and colleagues’ [20] population study (*N* = 1420) 29% of youth (age 9–13 years) had experienced a ‘recent stressor’ in the past 3 months. Research suggests that general life-stressors can contribute to youth internalizing problems [29–31]. Children with anxiety disorders tend to experience more stressful life events prior to onset of the disorder and perceive these events as more impactful compared to their non-anxious counterparts [28, 29, 32–35]. Life-stressors are also associated with youth depression [36–41]. Studies suggest that uncontrollable and unpredictable events, especially earlier in childhood, are relevant to the development of anxiety while undesirable major stressful events link to depression onset [8]. Although childhood adversity is a risk for internalizing, relatively little is known about how adversity exposure including its developmental timing influences mental health outcomes [42].

One of the strongest child-focused risk factors for internalizing problems is known in empirical literature to be an inhibited temperamental style [43, 44]. Inhibited temperament is the tendency for fearfulness/withdrawal in unfamiliar situations and is observed in 15–20% of children [45, 46]. Studies show that inhibited children are at greater risk of developing anxiety disorders than non-inhibited children [47–52]. Jaffee and colleagues [53] also found that child inhibition was a significant predictor of adolescent depression (odds ratio 3.09, *p* < .01). However, while inhibition is an important individual risk factor, not all children with this temperamental style develop internalizing problems.

Theory on ‘stress sensitization’ combines trauma and other life-stressors, posing that experiencing trauma early in life can sensitize individuals to having more negative reactions (such as internalizing symptoms) to other later life-stressors [54–56]. Therefore, individuals who experience childhood trauma seem to require less stress later to produce a reoccurrence of symptoms [35, 57]. McLaughlin, Conron, Koenen and Gilman’s [58] population study (*N* = 34,653 adults) found that exposure to childhood adversity plus stressful life events in the previous year predicted more internalizing problems for men and women, compared to prior year stressors without child adversity. One recent study of children focused on this theory. Grasso and colleagues [15] explored stress sensitization amongst preschool age children in disadvantaged United States neighbourhoods. In their sample of 213 clinically referred and non-referred young children, 58% experienced a traumatic event in their lifetime (age 4-years) while 38% had recent life-stressors in the past 3 months. Recent life-stressors were more frequent for those young children who had experienced trauma (46% vs. 33%; *p* < .05). Young children with the combination of traumatic events and recent life-stressors were at highest risk for internalizing problems, compared to those with trauma alone, recent stressors alone, or neither type of environmental stress.

In theory, temperamentally inhibited young children may be especially susceptible to environmental impacts due to their constitutional sensitivity to novelty/change [59]. They may be particularly sensitive to negative events in their environment. Consequently, inhibited preschool children may therefore be especially prone to stress sensitization if faced with a combination of early life trauma followed by later life-stressors. In stress-sensitization theory, a traumatic event early in life can sensitize individuals toward more negative responses to later life stresses. Therefore, it is important to know whether inhibited young children experiencing trauma in early childhood are especially vulnerable to internalizing difficulties when faced with life stressors later in childhood. To our knowledge this hypothesis has not been explored empirically.

A couple of studies with inhibited children have more generally explored risk for internalizing problems when encountering daily stressors, or a traumatic event (but not stress-sensitization). Previously, Brozina and Abela [60] explored whether inhibition predisposed school-age children (*N* = 384, age 8–13 years) to negative impacts of daily hassles. They found symptoms of anxiety increased significantly amongst children initially high in inhibition who then experienced high levels of daily hassles across 6 weeks. Otto and colleagues [61] explored whether early childhood inhibition was a risk for school-age children (*N* = 105, *M*_*age*_ 9-years) to develop posttraumatic stress symptoms after watching televised coverage of the 9/11 terrorist attacks. They found inhibited children were actually less likely to develop anxiety because they viewed less television after 9/11, thereby avoiding this potential stressor.

In sum, the mental health of young inhibited children may be especially sensitive to environmental impacts. Elucidating risk factors that further contribute to the development of internalizing disorders in this already vulnerable population can help clinicians to identify those who are most in need of early intervention. Therefore, this population study of inhibited preschool children aimed to explore whether those experiencing traumatic events in early childhood (6 months – 4 years) were especially vulnerable to later internalizing difficulties when faced with more general life-stressors in mid childhood (7–10 years). We expected inhibited children with early-life trauma would have more internalizing difficulties in mid childhood compared to those without early-life trauma. We predicted inhibited children with more general life-stressors recently in mid childhood would have higher internalizing difficulties. We then anticipated early-life trauma would sensitize inhibited young children to negative impacts of later general life-stressors, with the combination leading to highest mid-childhood internalizing difficulties.

## Methods

### Participants and procedure

This longitudinal study was part of a wider population-based prevention trial that commenced (2010) in metropolitan Melbourne (population 4 million) Victoria, Australia [62]. Ethics approval was by La Trobe University (HEDC13–002) and the Royal Children’s Hospital Melbourne (30105A). Eight of Melbourne’s 31 local government areas were selected to provide a broad socioeconomic spectrum (SEIFA) [63]. All government funded preschools in these districts were invited to take part. Across 2 years, participating preschools (*n* = 307, 78% uptake) distributed an inhibition screening questionnaire to all parents of children enrolled in their year before starting school (age 4-years). A total of 6347 parents returned their screening questionnaire of which 11% (*n* = 703) were inhibited and thereby eligible [64]. Parents with inhibited preschoolers were contacted by telephone and mailed the recruitment questionnaire and consent form. Of these, 545 (78%) parents consented to take part [65] and 455 (84%) later contributed data during the mid-childhood follow up (sample age 7–10 years). Children completed questionnaires in mid childhood only. In the first mid childhood follow up in 2015 (sample age 7–8 years), wave 1 parent questionnaires were completed by 82% (*n* = 446), parent interviews by 80% (*n* = 436) and child questionnaires by 74% (*n* = 403). In 2016 (age 8–9 years), wave 2 parent questionnaires were completed by 78% (*n* = 427), parent interviews by 77% (*n* = 418) and child questionnaires by 72% (*n* = 394). In 2017 (age 9–10 years), wave 3 parent questionnaires were completed by 78% (*n* = 426), parent interviews by 76% (*n* = 413) and child questionnaires by 73% (*n* = 399). Table 1 presents the sample characteristics. The sample recruited at preschool age represented the Australian population reasonably well (half girls, half first born, a quarter of parents born outside of Australia/New Zealand, parent education at all levels and a wide range of family income with hardship represented by a welfare card) [65]. In mid-childhood the sample retained was also representative across these domains.

**Table 1:** *Characteristics of Families with Inhibited Preschool Children at Recruitment and Mid Childhood*

Characteristic	Recruitment *(N* = 545)	Retained in mid childhood (*n* = 455)	*p*
*Child*			
Inhibition severity, *M* (*SD*)	34.2 (2.8)	34.1 (2.7)	0.640
Female, % (*n*)	48.3 (263)	47.9 (218)	0.914
Age (years), *M* (*SD*)	4.7 (0.4)	4.7 (0.4)	0.924
First born, % (*n*)	51.4 (280)	51.4 (234)	0.869
*Parents*			
Age (years), *M* (*SD*)			
Mother	37.5 (4.4)	37.9 (4.4)	0.586
Father	39.5 (4.9)	39.6 (4.9)	0.887
Relationship status, % (*n*)			0.987
Married/cohabiting	92.1 (502)	91.9 (418)	
Separated/divorced	6.2 (34)	6.4 (29)	
Single/widowed	1.7 (9)	1.8 (8)	
Born in Australia/New Zealand, % (*n*)			
Mother	73.0 (395)	74.1 (335)	0.764
Father	72.1 (365)	74.2 (313)	0.456
Highest level of education, % (*n*)			
Mother			0.795
Did not complete high school	11.4 (61)	11.6 (52)	
Completed high school	23.5 (126)	21.7 (97)	
Completed tertiary degree	65.2 (350)	66.7 (299)	
Father			0.924
Did not complete high school	18.2 (90)	17.4 (72)	
Completed high school	26.7 (132)	26.2 (108)	
Completed tertiary degree	55.2 (273)	56.4 (233)	
*Family*			
English main language at home, % (*n*)	88.6 (483)	90.3 (411)	0.383
Household income (AUD per annum, optional item), % (*n*)			0.978
< $25,000	4.6 (21)	4.7 (18)	
$25,000-51,900	12.9 (59)	11.9 (46)	
$52,000-88,400	22.8 (104)	22.6 (87)	
> $88,400	59.7 (273)	60.8 (234)	
Parent with welfare card, % (*n*)	18.7 (100)	16.6 (69)	0.447
Neighborhood disadvantage, *M* (*SD*)	1046.6 (42.6)	1046.8 (41.9)	0.942
All percentages are valid percent.			

### Measures

In the preschool age questionnaire, parents completed the Australian Temperament Project’s 7-item inhibition scale for screening eligibility (e.g., “*My child is shy with strange adults*”) [66]. This scale has sound psychometric properties with alpha .75 and significant continuity from infancy to school age [67, 68]. Item response options (1 = *almost never* to 6 = *almost always*) are summed and a score > 30 (85th percentile) classifies as inhibited [69]. At recruitment parents also provided family demographics and a neighborhood disadvantage score was assigned by home postcode (SEIFA, Australian *M (SD)* 1000 (100)) [70] (Table 1).

At each wave of mid childhood (annually, waves 1–3), recent life-stressors were measured by parent questionnaire. This asked if their child had experienced any on a list of 12 life-stressors in the past 3 months. The life-stressors were - new children in the home, parental separation/divorce, new parent figure, moving home, childcare change, reduction in standard of living, parent hospitalization, separation from parent for a week or longer, significant person moving away, death of pet, unsafe neighbourhood, loss of home. The list was as per Grasso et al. [15] which had been derived from the Preschool Age Psychiatric Assessment [71] and Child Life Events questionnaire [72]. For each child at each mid-childhood wave, the number of recent life-stressors was summed.

In addition, parents reported on any potentially traumatic events that had taken place over the course of their child’s lifetime in the final mid childhood questionnaire (wave 3). They were asked “Sometimes particularly stressful events have taken place in a child’s life. In addition to the [list of 12 stressors] above, if your child experienced a particularly stressful event (some time in their life) you can let us know here”. Examples were provided as illustration, “e.g., hospitalisation, car accident, animal attack, natural disaster, near drowning, fire, accidental burning/poisoning/serious fall, witness to death/violence, sudden death of a loved one”. These examples directly stemmed from Grasso et al.’s [15] measure that was derived from the PTSD section of the Preschool Age Psychiatric Assessment [71]. Parents were asked to indicate whether their child had experienced a particularly stressful event over the course of their life (yes/no) and if ‘yes’ to write the type of stressful event (open-ended), along with the child’s age when this took place. Events reported by parents in line with Grasso et al.’s potentially traumatic events were scored and the number in early childhood was summed (6 months to 4 years, as Grasso’s age-range for early trauma) [15].

At each mid childhood wave (waves 1–3), the Anxiety Disorder Interview Schedule, Child Version (ADIS) [73] was administered to parents by telephone. This covered child separation anxiety, social phobia, specific phobia and general anxiety disorders. The ADIS has been subjected to numerous reliability and validity studies and findings show it has good to excellent test-retest reliability, fair to excellent interrater reliability (*K* = .45–.82) and telephone administration has established validity with face-to-face interviews [74]. In the parent and child report questionnaires, the Spence Children’s Anxiety Scale (SCAS) [75] was included to measure anxiety symptoms. The SCAS covers symptoms of separation anxiety, social phobia, physical injury fears, obsessive compulsive, panic attack and agoraphobia, and generalised/overanxious disorder. Spence [75] described sound psychometrics for the SCAS, with internal consistency (α .92), split-half reliability (*r* .90), 6-month test-retest reliability (*r* .60), and convergent validity with the Revised Children’s Manifest Anxiety Scale (*r* .71) and Children’s Depression Inventory (*r* .48). The parent SCAS has 38 items and the child SCAS 44 items due to positive fillers. Item responses are 0 (*never true*) to 3 (*always true*) with the total SCAS score summed across items. The Short Moods and Feelings Questionnaire (SMFQ) [76] measured child depression symptoms by parent and child report. Angold et al. [76] describe sound psychometrics for the SMFQ including internal consistency (α .90), convergent validity with the Children’s Depression Inventory (*r* .67) and discriminant validity differentiating between clinical depression and non-clinical samples. The SMFQ has 13 items and response options (0 = *not true* to 2 = *true*) are summed to provide the total score.

### Analysis

Statistical analyses were performed using IBM SPSS version 23. Any relationship between early-life trauma and later mid-childhood stressors was first assessed by analysis of covariance (controlling for initial child inhibition severity and trial arm status). Each of the following analyses of variance controlled for the same two variables. The relationship between early-life trauma and mid-childhood internalizing symptoms (anxious and depressive) was assessed using multivariate analysis of covariance (MANCOVA). Chi square tests were used to assess the relationship between early-life trauma and mid childhood anxiety disorder. Then in mid-childhood, concurrent relations between recent life-stressors and internalizing symptoms were assessed using Pearson partial correlations (with control for the same two variables). The relationship between recent life-stressors and child anxiety disorder was assessed by ANCOVA. Last, inhibited children were divided into four groups: early trauma plus recent life-stressors, early trauma without recent stressors, recent stressors without early trauma, and neither early trauma nor recent stressors. MANCOVA compared the four groups on mid-childhood internalizing symptoms and chi-square compared these groups on anxiety disorder.

## Results

In this population sample of inhibited preschool children, 29 (6%) had experienced a potentially traumatic event in the first 4 years of life, of whom 83% experienced one traumatic event (*n* = 24) and 17% experienced two traumatic events (*n* = 5). The types of potentially traumatic events experienced by inhibited preschoolers in the general population were - child hospitalization (41%, *n* = 12), death of a loved one (38%, *n* = 11), parent life threatening illness (14%, *n* = 4), natural disaster (7%, *n* = 2), parent abandoned child (3%, *n* = 1), parent removed from home (3%, *n* = 1), sibling life threatening illness (3%, *n* = 1), car accident (3%, *n* = 1), and animal attack (3%, *n* = 1).

Later in mid childhood, 256 (56%) of the children in this general population sample of inhibited pre-schoolers experienced broader recent life-stressors (Table 2). Most commonly this was separation from a parent for a week or longer, reported for one in 10 inhibited children. The other more common life-stressors were moving home, death of a pet, parent hospitalization, or a significant person moving away. Report of early-life trauma was not associated with number of recent life-stressors in mid-childhood (ANCOVAs: **wave 1:***F* (1,405) =0.00, *p* = 0.948; **wave 2:***F* (1,404) =0.28, *p* = 0.559; **wave 3:***F* (1,411) =0.04, *p* = 0.836). The group of children with early trauma did not differ significantly from the group with later life stressors on demographic variables in Table 1, apart from the proportion of children with single or widowed parents (21% in early trauma group vs 8% in later life stressors group, *p* = 0.037).

**Table 2:** *Recent Life Stressors Experienced by Inhibited Children in Mid Childhood*

Type of stressor, % (*n*)	Wave 1 (2015) (age 7–8 years)	Wave 2 (2016) (age 8–9 years)	Wave 3 (2017) (age 9–10 years)
Separation from parent for a week or longer	9.9 (44)	11.5 (49)	11.3 (48)
Moving home	4.9 (22)	6.8 (29)	7.5 (32)
Death of a pet	6.3 (28)	4.2 (18)	6.1 (26)
Parental hospitalization	4.7 (21)	5.4 (23)	3.3 (14)
Significant person moving away	3.8 (17)	4.5 (19)	3.5 (15)
Childcare change	5.0 (22)	3.8 (16)	2.6 (11)
Parental separation/divorce	3.1 (14)	4.2 (18)	1.6 (7)
Reduction in standard of living	2.0 (9)	2.1 (9)	3.1 (13)
New children in home	2.5 (11)	2.6 (11)	1.6 (7)
Unsafe neighborhood	1.4 (6)	2.6 (11)	1.2 (5)
New parent figure	2.0 (9)	1.2 (5)	1.4 (6)
Loss of home	0.5 (2)	0.2 (1)	0.7 (3)

All percentages are valid percent

Relationship between early-life trauma and mid-childhood internalizing symptoms (anxious and depressive) was assessed by MANCOVA for parent and child report at each wave (see Table 3). Inhibited preschoolers with and without early-life trauma differed on later mid-childhood internalizing symptoms in two of the three waves by parent report, with small effect sizes (Table 3, partial η^2^ = .02) [77]. Subsequent ANCOVAs (see Table 4) showed that the inhibited children with early trauma had more anxious but not depressive symptoms, than those without early trauma in mid childhood in these waves. The inhibited children with early trauma also had more anxiety disorder in one wave than those without early trauma (**wave 1**: 52% vs. 41%, χ^2^(1, *n* = 405) =1.35, *p* = 0.245; **wave 2**: 55% vs. 36%, χ^2^(1, *n* = 402) =4.39, *p* = 0.036; **wave 3**: 38% vs. 27%, χ^2^(1, *n* = 403) =1.52, *p* = 0.218). However, the child report MANCOVA showed no relationship between early trauma and mid childhood internalizing difficulties.

**Table 3:** *Early Life Trauma and Mid Childhood Internalizing Symptoms: MANCOVA results*

Mid childhood wave	*df*	*F*	Wilks’ λ	*p*
*Parent report*				
Wave 1 (age 7–8 years)	2, 403	2.34	0.99	0.097
Wave 2 (age 8–9 years)	2, 400	4.12	0.98	0.017
Wave 3 (age 9–10 years)	2, 409	5.07	0.98	0.007
*Child report*				
Wave 1 (age 7–8 years)	2, 368	1.59	0.99	0.206
Wave 2 (age 8–9 years)	2, 372	0.41	1.00	0.665
Wave 3 (age 9–10 years)	2, 380	0.74	1.00	0.477

Controlling for preschool inhibition severity and prevention trial arm status

**Table 4:** Subsequent ANCOVA Results for Early Life Trauma and Mid Childhood Internalizing Symptoms

Internalizing scale (parent report), *M (SE)*	Early trauma (*n* = 29)	No early trauma (*n* = 387)	*F*	*p*
Anxiety (SCAS)				
Wave 2 (age 8–9 years)	26.01 (2.35)	19.15 (0.65)	7.92	0.005
Wave 3 (age 9–10 years)	25.82 (2.34)	18.11 (0.64)	10.15	0.002
Depression (SMFQ)				
Wave 2 (age 8–9 years)	3.65 (0.75)	2.73 (0.21)	1.40	0.238
Wave 3 (age 9–10 years)	3.55 (0.69)	2.43 (0.19)	2.46	0.118

Controlling for preschool inhibition severity and prevention trial arm status

*SCAS* Spence Children’s Anxiety Scale, *SMFQ* Short Moods and Feelings Questionnaire

Life-stressors in mid childhood concurrently related to inhibited children’s internalizing difficulties. Anxiety symptoms showed small positive correlations by parent and child report in two of the three waves (**wave 1:** parent report, *r* = 0.07, *p* = 0.151; child report, *r* = 0.10, *p* = 0.064) (**wave 2:** parent report, *r* = 0.18, *p* > 0.001; child report, *r* = 0.11, *p* = 0.035) (**wave 3:** parent report, *r* = 0.13, *p* = 0.007; child report, *r* = 0.13, *p* = 0.011). Depression symptoms showed small positive correlations by parent report in the second wave and child report in the third wave (**wave 1:** parent report, *r* = 0.06, *p* = 0.176; child report, *r* = 0.06, *p* = 0.243) (**wave 2:** parent report, *r* = 0.15, *p* = 0.002; child report, *r* = 0.10, *p* = 0.060) (**wave 3:** parent report, *r* = 0.09, *p* = 0.077; child report, *r* = 0.12, *p* = 0.018). ANCOVA compared recent life-stressors for children with and without anxiety disorder. Inhibited children with anxiety disorder experienced more recent life-stressors than those without anxiety disorder at one wave (**wave 1:***M* (*SD*) 0.45 (0.76) vs*,* 0.46 (0.90), *F* (1,426) = 0.01, *p* = 0.962) (**wave 2:***M* (*SD*) 0.68 (1.14) vs. 0.39 (0.77), *F* (1,413) = 10.31, *p* = 0.001) (**wave 3:***M* (*SD*) 0.47 (0.84) vs. 0.41 (0.79), *F* (1,408) = 0.91, *p* = 0.341).

MANCOVA then tested for stress sensitization, comparing internalizing symptoms for the four groups - early trauma plus recent stressors, early trauma only, recent stressors only, or neither type of environmental stress. There was a difference between the four groups on child internalizing symptoms by parent report in the second and third wave (see Table 5 for details). Subsequent ANCOVAs (see Table 6) indicated a difference between the four groups on anxiety and depression symptoms at those waves. However, inhibited children with both early trauma and recent life-stressors did not have the highest internalizing symptoms. Rather, children with early trauma only, or recent life-stressors only, had more internalizing symptoms than the group without trauma or stressors (see Table 6). Likewise, there were no differences in anxiety disorder between the four groups to indicate stress sensitization (trauma plus stress, stress only, trauma only, neither) (**wave 1:** 58% vs. 42% vs. 47% vs. 40%, Fisher’s exact test =2.04, *p* = 0.554) (**wave 2:** 50% vs. 43% vs. 59% vs. 33%, Fisher’s exact test =7.96, *p* = 0.045) (**wave 3:** 33% vs. 29% vs. 40% vs. 27%, Fisher’s exact test =1.82, *p* = 0.611).

**Table 5:** Four Groups with Different Combinations of Early Life Trauma and Recent Life Stressors: MANCOVA results

Mid childhood internalizing symptoms	*df*	*F*	Wilks’ λ	*p*
*Parent report*				
Wave 1 (age 7–8 years)	6, 802	1.49	0.98	0.176
Wave 2 (age 8–9 years)	6, 798	3.58	0.05^a^	0.002
Wave 3 (age 9–10 years)	6, 814	3.58	0.51^a^	0.002
*Child report*				
Wave 1 (age 7–8 years)	6, 732	1.64	0.97	0.133
Wave 2 (age 8–9 years)	6, 740	1.38	0.98	0.219
Wave 3 (age 9–10 years)	6, 756	1.30	0.02^a^	0.254

Controlling for preschool inhibition severity and prevention trial arm status

^a^Pillai’s trace is reported rather than Wilks’ λ (Box’s M indicated violation of the assumption of homogeneity of covariance)

**Table 6:** Subsequent ANCOVA Results for Four Groups with Different Combinations of Early Life Trauma and Recent Life Stressors

Parent report, *M* (*SE*), *n*	Neither	Early trauma only	Recent stress only	Early trauma and recent stress	*F*	*p*
Anxiety (SCAS)						
Wave 2 (age 8–9 years)	17.80 (0.76), 270	22.60 (1.21), 17 *	28.25 (3.03), 106 *	22.81 (3.60), 12	6.88	< 0.001
Wave 3 (age 9–10 years)	17.05 (0.75), 276	20.90 (1.21), 20 *	26.00 (2.79), 108 *	25.41 (4.17) 9	5.82	0.001
Depression (SMFQ)						
Wave 2 (age 8–9 years)	2.37 (0.25), 270	3.64 (0.39), 17	4.11 (0.98), 106 *	3.01 (1.16), 12	3.16	0.025
Wave 3 (age 9–10 years)	2.21 (0.23), 276	2.97 (0.36), 20	2.80 (0.83), 108	5.24 (1.24), 9	2.77	0.041

Controlling for preschool inhibition severity and prevention trial arm status

*SCAS* Spence Children’s Anxiety Scale, *SMFQ* Short Moods and Feelings Questionnaire

*p < 0.05, significant difference compared to ‘Neither’ group

## Discussion

In stress sensitization theory, a traumatic event in early life may sensitize individuals toward more negative responses to later life-stressors [54–56]. In temperament theory, inhibited children can be particularly sensitive to the effects of their environment [59]. Therefore, this population-level study of inhibited preschool children explored whether those experiencing traumatic events in early childhood were especially vulnerable to later internalizing difficulties when faced with more general life-stressors in mid childhood. Six percent of inhibited preschoolers in the Australian context (one in every 14) experienced a potentially traumatic event in their first 4 years of life. Later in mid childhood, 60% of the inhibited population sample experienced broader life-stressors. Those inhibited children with early-life trauma developed slightly more mid-childhood anxiety symptoms and clinical disorder (parent source). Those facing higher general life-stressors in mid-childhood also had slightly more anxious and depressive symptoms (parent and child sources). However, in regard to stress sensitization, those inhibited children with the *combination* of early-life trauma and broader recent life-stressors did not show the most mid-childhood internalizing problems.

Although stress-sensitization effects were not detected for inhibited children in the Australian general population, our findings (as in the United States) [21] indicated higher anxiety symptoms in children exposed to traumatic events. However, our sample had comparatively low levels of exposure to traumatic events, and even lower levels of exposure to multiple traumas. It could be that multiple severe traumas are the level to induce stress sensitization [78, 79].

A reason for the lower level of traumatic events in our sample may have to do with economic factors. Compared to the general Australian population, traumatic events experienced by young children in the United States (USA) might be higher due to economic disparity [80, 81]. In the USA, 25–31% youth (spanning childhood and adolescence) in the general population experience potentially traumatic events [19, 20]. In disadvantaged locations trauma rates for very young American children are also at this rate (26%) [21]. It is possible that our sample had lower rates of traumatic events than the samples included in US studies of sensitization because of economic disparities between those samples and ours. Briggs-Gowan and Grasso’s [15, 21] sample included 45% of families in poverty and severe trauma (family violence, burns, serious falls, poisonings). Their stress sensitization finding for young children was with clinically referred and non-referred children from poor USA neighborhoods (45% poverty, 48% in treatment). Two thirds experienced early childhood trauma, of which a third were multiple traumatic events (up to four). Our population sample of inhibited preschoolers in Australia, by comparison, had experienced fewer traumatic events in early childhood (6%, usually one and occasionally two events). Stress sensitization may take place only with early severe and prolonged adversity. Perhaps in line with this view, a study of adults by McLaughlin et al. [58] found 3+ ‘adverse childhood experiences’ forecast adult internalizing problems whereas 0–2 did not (although this was not restricted to trauma).

It is also possible that inhibited young children have lower trauma exposure because of their cautious, novelty- and risk-avoidant behavioral style, together with that of their parents (biological relatives, due to genetic factors). Along this line, children with attention deficit hyperactivity disorder (ADHD, extreme behavioral disinhibition) tend to experience more traumatic events than those without this disorder [82]. For clarification, rates of trauma would need to be compared between inhibited and non-inhibited children in epidemiological samples.

Interestingly, the number of broader life-stressors experienced by inhibited children in mid-childhood seems higher than population rates for children in general. Costello and colleagues [20] reported that 29% of youth aged 9–13 years experienced recent stressful events in the past 3 months. In our inhibited sample 56% of children aged 7–10 years experienced recent life-stressors in the past 3 months. Temperamentally inhibited children do tend to experience a variety of daily-life circumstances to be stressful in comparison to their uninhibited peers. Also consistent with other international studies, we found that more mid-childhood life-stressors was associated with more child internalizing problems [28–35, 37]. It is clear that temperamentally inhibited children facing general life-stressors are at some risk for developing internalizing problems.

The present study had the following limitations. Families facing high adversity may have chosen not to participate in the initial population trial. However, the sample’s demographics show that many families facing risks were recruited. While tertiary educated parents and families with more income were over-represented, still 12% of parents had not completed high school and one in five families had a welfare card that indicates financial hardship. The sample included a wide range of parent education and family income (including lower incomes^1^), along with diversity in cultural backgrounds. Longitudinal retention was high and those retained in mid childhood were sociodemographically comparable. The study findings can therefore be generalised to a wide socioeconomic spectrum, although perhaps not the lowest end [65]. Another limitation is that retrospective report by parents of traumatic events in their child’s life relies on memory. Conceptualisation of early trauma and recent life-stressors in our study aimed for consistency with definitions in Grasso et al.’s early childhood study [15]. While both included retrospective report of potentially traumatic events, recall in Grasso et al.’s study [15] was over a shorter period of time. It may be that some traumatic events that took place in inhibited young children’s lives were forgotten by their parents over time in the present study.

With these limitations in mind, findings of this study could have some positive clinical implications. Many parents with inhibited young children express concerns about whether some negative event ‘caused’ their child to be highly anxious. Clinicians may therefore be able to offer reassurance to many parents with inhibited youngsters that it is relatively uncommon for children of this temperamental style to have experienced traumatic events. Further, for those that have, effects on their mental health in the longer term appear to be small. Guidance could be offered to foster resilience. Children can model distress behaviors by observing parents’ own responses to life stressors [10, 12, 83–87]. For parents to maintain calm and positive parenting could therefore be protective when their inhibited child is faced with stressors [10]. Early prevention programs have been developed to facilitate inhibited children’s resilience to life stressors as they grow. Cool Little Kids, for example, guides parents in responding to their young child’s emotional distress in ways that develop positive coping skills [69]. Buthmann and colleagues note a need for deeper understanding of interactions between parental mood and temperamental reactivity of the developing child that could be explored in future early intervention research for internalizing [88].

## Conclusions

This study provides insight for pediatric clinicians who see shy/inhibited young children in general practice. The findings provide new knowledge about the types of environmental stress that tend to be experienced by temperamentally sensitive children in the general population. A small proportion of inhibited young children do experience potentially traumatic events in early life. However, in mid-childhood many more inhibited children (two thirds) face broader life-stressors. Each of early-life trauma and concurrent life-stressors may convey some small risk for an inhibited child to develop internalizing difficulties. In the general population nevertheless, stress sensitization is not common place for inhibited young children. Future research can explore how parenting practices together with parent skills in mood regulation could be a source of resilience to inhibited children in navigating stressful events that will be encountered in their lives.

## Data Availability

The datasets generated and/or analysed during the current study are not publicly available due to privacy or ethical restrictions but are available from the corresponding author on reasonable request.

## Footnotes

1 Australian median family income for couples with dependent children (eldest < 5 years) $103,688 (Australian Bureau of Statistics 2011–2012 Household Survey).
